# Cardiorespiratory fitness and mortality risk in patients receiving hemodialysis: a prospective cohort

**DOI:** 10.1590/2175-8239-JBN-2022-0124en

**Published:** 2023-07-21

**Authors:** Francini Porcher Andrade, Carolina Ferraro Borba, Heitor Siqueira Ribeiro, Paula Maria Eidt Rovedder

**Affiliations:** 1 Universidade Federal do Rio Grande do Sul, Ciências Pneumológicas Post-Graduation Program, Porto Alegre, RS, Brazil.; 2 Universidade Federal do Rio Grande do Sul, Escola de Fisioterapia, Porto Alegre, RS, Brazil.; 3 Universidade de Brasília, Faculdade de Ciências da Saúde, Brasília, DF, Brazil.

**Keywords:** Renal Insufficienc, Chroni, Renal Dialysi, Cardiorespiratory Fitnes, Peak Oxygen Uptak, Mortality, Insuficiência Renal Crônica, Diálise Renal, Aptidão Cardiorrespiratória, Consumo de Oxigênio de Pico, Mortalidade

## Abstract

**Background:**

Kidney failure reduces life expectancy by one-third compared with the general population, and cardiovascular complications and poor cardiorespiratory fitness (CRF) are the main causes. We aimed to evaluate the association between severely low CRF and all-cause mortality risk in HD patients.

**Methods:**

This observational prospective cohort study followed-up patients receiving HD from August 2015 until March 2022. Cardiorespiratory fitness was evaluated through the cardiopulmonary exercise test, and the peak oxygen uptake (VO_2peak_) value was used to determine severely low CRF (< 15 mL∙kg^−1^∙min^−1^). Cox regression and univariate Kaplan-Meier analysis were used to evaluate the association of severely low CRF with mortality risk and survival rate.

**Results:**

Forty-eight patients were followed-up for a median of 33.0 [14.3 – 49.3] months. A total of 26 patients had severely low CRF. During the follow-up period, 11 patients (22.92%) died from all causes. From these, eight (30.8%) had severely low CRF. Even so, severely low CRF was not associated with crude death rates for patients stratified by CRF levels (p = 0.189), neither in unadjusted (HR 2.18; CI 95% 0.58−8.23) nor in adjusted (HR 1.32; CI 95% 0.31−5.59) Cox proportional hazard models. As a continuous variable, VO_2peak_ was not associated with mortality risk (HR 1.01; CI 95% 0.84−1.21). Univariate Kaplan-Meier analysis showed that patients with severely low CRF did not have significantly worse survival rates than those with mild-moderate CRF (p = 0.186).

**Conclusion:**

Our findings indicated that severely low CRF was not associated with all-cause mortality in patients on HD. Despite severely low CRF being prevalent, larger cohort studies are needed to establish strong conclusions on its association with all-cause mortality.

## RESUMO

**Introdução:**

A insuficiência renal reduz a expectativa de vida em um terço comparada à população em geral. Complicações cardiovasculares e baixa aptidão cardiorrespiratória (ACR) são as principais causas. Avaliamos a associação entre ACR muito baixa e risco de mortalidade por todas as causas em pacientes em HD.

**Métodos:**

Este estudo de coorte prospectivo observacional acompanhou pacientes em HD de agosto/2015 a março/2022. Avaliou-se a aptidão cardiorrespiratória pelo teste de exercício cardiopulmonar, e o valor do pico do consumo de oxigênio (VO_2pico_) foi usado para determinar ACR muito baixa (< 15 mL∙kg−1∙min−1). Utilizamos regressão de Cox e análise univariada de Kaplan-Meier para avaliar associação da ACR muito baixa com o risco de mortalidade e taxa de sobrevida.

**Resultados:**

Acompanhamos 48 pacientes por uma média de 33,0 [14,3 – 49,3] meses. Um total de 26 pacientes apresentaram ACR muito baixa. No período de acompanhamento, 11 pacientes (22,92%) foram a óbito por todas as causas. Destes, oito (30,8%) apresentavam ACR muito baixa. Mesmo assim, ACR muito baixa não foi associada a taxas brutas de mortalidade para pacientes estratificados por níveis de ACR (p = 0,189), nem em modelos de risco proporcional de Cox não ajustados (HR 2,18; IC 95% 0,58–8,23) ou ajustados (HR 1,32; IC 95% 0,31–5,59). Como variável contínua, VO2pico não foi associado ao risco de mortalidade (HR 1,01; IC 95% 0,84–1,21). A análise univariada de Kaplan-Meier mostrou que pacientes com ACR muito baixa não apresentaram taxas de sobrevida significativamente piores do que aqueles com ACR leve-moderada (p = 0,186).

**Conclusão:**

Nossos achados indicaram que a ACR muito baixa não foi associada à mortalidade por todas as causas em pacientes em HD. Apesar de ACR muito baixa ser prevalente, são necessários estudos de coorte maiores para estabelecer conclusões sólidas sobre sua associação com mortalidade por todas as causas.

## Introduction

Kidney failure reduces life expectancy by one-third compared with the general population^1^. Different factors are associated with morbidity and mortality in chronic kidney disease (CKD) patients, mainly those in hemodialysis (HD), such as cardiovascular complications and poor cardiorespiratory fitness (CRF)^2,3,4,5,6^. CRF is affected by both CKD and HD treatment and is strongly related to a wide spectrum of health outcomes, including poor cardiovascular health^2^.

CRF undergoes a physiological drop with aging. As shown by Imboden et al.^7^, a decline in peak of oxygen uptake (VO_2peak_) of 1-MET per decade was observed in healthy individuals. In addition to aging, patients undergoing HD may have poor CRF due to the sedentary pattern and exercise limitation that are common in patients on HD^8^. Martins et al.^9^ performed a systematic review of observational studies and found a significant reduction in all-cause mortality with increased levels of physical activity in patients with CKD. In addition, previous research proposed that both an increase in physical activity level^10^ and CRF^11^ and a reduction in morbidity^12^ in these patients may be reached through exercise programs.

CRF is mainly evaluated through a cardiopulmonary exercise test (CPET), which is lab-based and considered the gold standard. The CPET provides an objective determination of CRF through direct measurement of VO_2peak_^13^. Patients receiving HD commonly show lower VO_2peak_ than healthy individuals^14^ and this lower CRF seems to be a strong predictor of mortality^5^.

According to Sietsema et al.^5^, a higher mortality rate is seen in patients receiving HD with VO_2peak_ values < 17.5 mL∙kg^−1^∙min^−1,5^. Even so, no recent study has explored this association and there is a need to understand this phenomenon in order to develop future strategies to prevent or treat cardiovascular complications associated with poor CRF, such as mortality. In this sense, measures of exercise capacity that reflect cardiovascular health may be important for risk assessment in these patients. Therefore, this study aimed to evaluate the association between severely low CRF and VO_2peak_ values with all-cause mortality in patients receiving HD.

## Methods

### Data Source and Study Population

This was an observational prospective cohort study that evaluated CKD patients undergoing conventional HD treatment. Patients were eligible for inclusion if they were aged >18 years, on HD ≥3 months, and had clinical stability (i.e., absence of hospitalization in the previous 30 days). Exclusion criteria were acute myocardial infarction within 3 months, inflammatory process under treatment with anti-inflammatory or antibiotic drugs in the previous 30 days, decompensated coronary artery disease, symptomatic peripheral arterial disease, arterial access in the lower limbs, musculoskeletal impairment, and serum hemoglobin level < 10.0 g/dL (100 g/L). The study was approved by the Ethics and Research Committee of Hospital de Clínicas de Porto Alegre (HCPA), under the number CAAE 40167014.3.0000.5327. Written informed consent was obtained according to the Declaration of Helsinki. This study is reported as per the Strengthening the Reporting of Observational Studies in Epidemiology (STROBE) checklist.

### Follow-Up Period

In March 2022, approximately 79 months after the baseline assessments, the researchers consulted the medical record of the patients to ascertain survival status and date of death.

### Measurements

#### Data collection

Demographic data including age, sex, smoking habits, weight, body mass index (BMI), and use of beta-blockers medication were collected. In addition, patients were asked about their exercise routine twice or more times a week. Dialysis-related factors, including the cause of CKD and HD vintage, were also collected.

#### Cardiopulmonary exercise test

CPET was performed on a non-dialysis day using a cycle ergometer to evaluate the relative VO_2peak_ (mL∙kg^−1^∙min^−1^), ventilation (VE), oxygen (O_2_) pulse, CPET duration (minutes), CPET work rate (Watts [W]), respiratory rate (RR), and heart rate (HR) on VO_2peak_. The test was applied with an incremental load of 5 or 10 W per minute^13^. The incremental load protocol was defined by the authors according to the American Thoracic Society (ATS) and the American College of Chest Physicians (ACCP)^13^ guidelines and according to CKD cause. Those with suspicion of hypertension as CKD cause were submitted to a 5-W increment due to possible hemodynamic and cardiovascular acute adverse events. Subjects with other CKD causes had a 10-W increase protocol. In addition, all patients were instructed to maintain their routine medications, such as beta-blockers and vasodilators.

CPET was performed in the Vmax^®^ Encore metabolic cart system (CareFusion, San Diego, California, USA) using a gas analyzer. A 10-lead CardioSoft electrocardiogram was used to evaluate the heart electrical function. The subjects were also monitored during the whole CPET through pulse oximetry to obtain oxygen saturation, and a manual sphygmomanometer in the non-fistulated arm was used to obtain the blood pressure. Arterial pressure, dyspnea perception, and lower limb fatigue (evaluated by the Borg CR10 Scale) were constantly registered^13,15^. Patients were verbally encouraged by physiotherapists before and during the whole CPET to obtain a maximum physiological exertion (respiratory exchange ratio [RER] >1.0)^13^. CPET consisted of 4 phases: a) three-minute rest to verified the absence of hyperventilation; b) a warm-up unloaded cycling (0 W for two-minute); c) an exercise phase with increments every minute (5/10 W – cycling rate at 60–65 revolutions per minute) until the subject signals to stop the test because of volitional exhaustion associated with an RER >1.0 or the test is ended by the medical professional. If the subject did not reach an RER >1.0, they were encouraged to continue the test; d) an active recovery phase, unloaded (0 W for one-minute). CPET was interrupted as suggested by the ATS/ACCP under the supervision of a physician^13^.

#### Cardiorespiratory fitness level

Patients receiving HD were separated into two groups according to their CRF levels, which were determined using the VO_2peak_ values according to the ATS suggestion. Patients with VO_2peak_< 15 mL∙kg^−1^∙min^−1^ were considered as severely low CRF and were defined as the exposure group. Mild-moderate CRF was considered for patients with VO_2peak_ ≥15 mL∙kg^−1^∙min^−117,16^.

### Statistical Analyses

Continuous data are presented as median and interquartile range or mean and standard deviation, depending on data normality. Categorical data are shown as frequencies and percentages. The Kolmogorov-Smirnov test was used to check continuous data for normality. Comparisons among the VO_2peak_ groups were conducted using the Wilcoxon Mann-Whitney test or the independent Student t-test for continuous variables. Categorical data were compared using the Chi-Square or Fisher’s exact tests.

Fisher’s exact test was used to compare crude death rates, and univariate survival analyses were performed using the log-rank test on survival curves created with the Kaplan-Meier method. Survival data were not censored at the time of kidney transplantation. To investigate the association between severely low CRF and all-cause mortality, time-to-event data were considered. The Cox proportional hazard model with a 95% confidence interval (CI) was calculated, and survival data were also not censored at the time of kidney transplantation. Patients with mild-moderate CRF were considered the reference group. Potential confounders (age, gender, BMI, and HD vintage) were added in the adjusted model^18^. Due to the small sample size, no sensitivity analysis was carried out. All analyses were performed using the Statistical Package for the Social Sciences (version 26.0, SPSS Inc, Chicago, USA) and GraphPad Prism (version 8, GraphPad Software, San Diego, USA). In addition, the sample power calculation for mortality ratio was performed using the PSS Health online version^19^. A p-value <0.05 was considered statistically significant.

## Results

### Baseline and Follow-Up Characteristics

Sixty-one patients on HD were assessed for eligibility criteria. Eight patients declined to participate, two patients had lower limb vascular access, two patients had decompensated coronary arterial disease and one patient was lost to follow-up. Therefore, 48 patients on HD were evaluated and followed-up from August, 2015 until March, 2022 for a median of 33.0 [interquartile range: 14.3 – 49.3] months. In addition, the patients were separated into two groups according to their CRF levels, and baseline characteristics are shown in Table 1. All patients used beta-blocker medication. Only one patient had the CPET interrupted by the physician due to ischemic electrocardiographic abnormalities. This interruption occurred after reaching an RER >1.0.

**Table 1.** General characteristics of the patients receiving hemodialysis according to CRF level

Characteristic	All patients (n = 48)	Severely low CRF (n = 26)	Mild-moderate CRF (n = 22)	p-value
**Demographics and Clinical**				
Age (years)	53.7 ± 15.2	60.5±11.4	45.7 ± 15.5	**< 0.001**
Elderly, *n* (%)	18 (37.5)	13 (50.0)	5 (22.7)	0.052
Female, *n* (%)	21 (43.8)	10 (38.5)	11 (50.0)	0.561
Hemodialysis vintage (months)	18.5 [6.8 – 74.5]	23.0 [8.8-79.3]	9.0 [5.3 – 70.0]	0.131
Smoking, *n* (%)^a^	26 (54.2)	19 (73.1)	7 (31.8)	**0.009**
Self-reported exercise routine	28 (58.3)	12 (46.2)	16 (72.7)	0.063
**Causes of chronic kidney disease,** ***n*** **(%)**				0.256
Glomerulonephritis	10 (20.8)	4 (15.4)	6 (28.6)	
Diabetes mellitus	7 (14.6)	6 (23.1)	1 (4.8)	
Hypertension	11 (22.9)	7 (26.9)	4 (19.0)	
Lupus	5 (10.4)	3 (11.5)	2 (9.5)	
Others	7 (14.6)	1 (3.8)	6 (27.3)	
Unknown	7 (14.6)	5 (19.2)	2 (9.5)	
**Body Composition**				
Weight (kg)	76.2 ± 15.6	79.9 ± 15.7	71.9 ± 14.6	0.075
Body mass index (kg/m^2^)	27.3 ± 4.3	28.3 ± 4.4	26.1 ± 4.0	0.090

CRF: cardiorespiratory fitness. Data are expressed as number and frequency (%), mean ± standard deviation (parametrical data), or median and [interquartile range] (non-parametrical data). ^a^n = 46 due to missing values.

Patients with severely low CRF were older (60.5 ± 11.4 *versus* 45.7 ± 15.5, p < 0.001) and more of them were smokers (73.1% *versus* 31.8%, p = 0.009). In addition, according to the self-reported exercise routine, a total of 58.3% of patients were enrolled in an intradialytic exercise program or attended a sports center during the follow-up period two or more times a week. Although there was an expressive difference between the groups (72.7% versus 46.2%), the comparison by self-reported exercise routine did not show a significant difference (p = 0.063), as shown in Table 1.

Table 2 shows the difference in CRF variables between groups. Severely low CRF patients had worse respiratory performance on CPET evaluated through VE (p < 0.001) and RR (p < 0.001), worse exercise tolerance evaluated through the CPET duration (p = 0.022) and final work rate (p < 0.001), and worse cardiac performance evaluated through HR on VO_2peak_ (p = 0.001) and O_2_ pulse predict value (p = 0.012). The CR10 Borg scale for lower limb fatigue (p = 0.535) and dyspnea (p = 0.451) were not different between groups. During the follow-up period, 21 patients (43.7%) remained in HD treatment, 16 patients (33.3%) were transplanted, and 11 patients (22.9%) died of all causes.

**Table 2.** Cardiorespiratory fitness according to CRF level

	All patients (n = 48)	Severely low CRF (n = 26)	Mild-moderate CRF (n = 22)	p-value
VO_2peak_ (mL∙kg^−1^∙min^−1^)	14.1 [13.2 – 18.6]	13.4 [11.5 – 13.8]	18.9 [16.5 – 23.3]	< 0.001
VO_2peak_ predicted (%)	64.2 [54.9 – 81.9]	59.0 [52.0 – 64.6]	81.9 [64.2 – 90.9]	< 0.001
VE (L)	46.02 ± 15.52	38.34 ± 9.84	55.09 ± 16.27	< 0.001
VE predicted (%)	58.05 ± 16.76	51.06 ± 10.13	66.31 ± 19.36	0.001
O_2_ pulse	9.7 [7.3 – 11.8]	8.9 [6.9 – 11.6]	11.05 [8.8 – 11.8]	0.146
O_2_ pulse predicted (%)^a^	92.30 ± 23.53	85.08 ± 19.19	100.50 ± 25.69	0.012
CPET duration (min)	10 [8 – 14]	9 [7 – 13]	12 [10 – 15]	0.022
Final work rate (watts)	72.4 ± 32.9	55.2 ± 21.8	92.7 ± 32.6	< 0.001
Work rate predicted (%)	52.37 ± 17.92	42.35 ± 11.63	64.21 ± 16.94	< 0.001
RER	1.11 ± 0.8	1.08 ± 0.06	1.15 ± 0.1	0.017
RR	31.5 [26.0 – 39.7]	26.5 [24 – 32.5]	34 [31.7 – 41]	< 0.001
VO_2peak_ heart rate (bpm)	123.1 ± 27.9	111.0 ± 23.6	137.4 ± 26.1	0.001
Borg lower limb fatigue (score)	9[5.5 – 10]	8[5 – 10]	9[6 – 9]	0.535
Borg dyspnea (score)	5 [2 – 8]	7 [1 – 9]	5 [2 – 7]	0.451

CRF: cardiorespiratory fitness; VO_2peak_: peak oxygen uptake; VE: ventilation; O_2_ pulse: oxygen pulse; CPET: cardiopulmonary exercise testing; RER: respiratory exchange ratio; RR: respiratory rate. Data are expressed as mean ± standard deviation (parametrical data), or median and [interquartile range] (non-parametrical data). ^a^n = 46 due to missing values.

### Association Between CRF and Mortality

**Figure 1.**
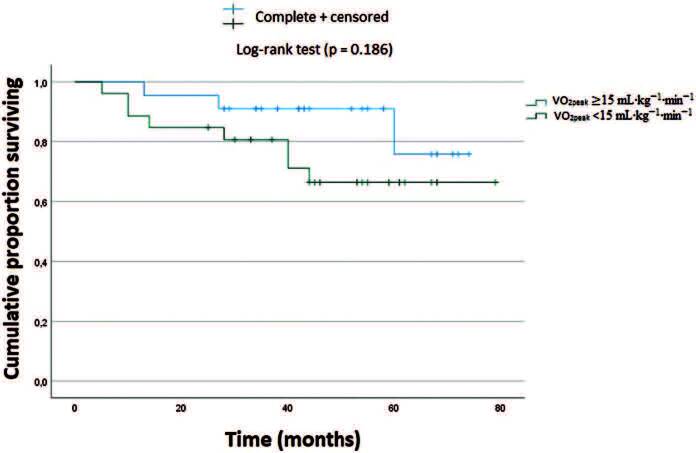
Survival analysis of all-cause mortality according to the CRF level.

Eight patients (30.8%) with severely low CRF died during the follow-up period; Fisher’s exact test showed an absence of statistical significance in the comparison of the crude death rates for patients stratified by CRF levels (p = 0.189). In addition, the univariate Kaplan-Meier analysis (Figure 1) showed that patients with severely low CRF did not have a significantly worse survival rate than those with mild-moderate CRF (p = 0.186). Cox proportional hazard model showed that severely low CRF was not associated with all-cause mortality in both unadjusted (HR 2.18; 95% CI 0.58−8.23) and adjusted models (HR 1.32; CI 95% 0.31−5.59). A 14.6% sample power was achieved to test whether the mortality ratio in patients with severely low CRF is different from 30.8%. This result (calculated by the exact method) was obtained considering a significance level of 5%, a sample size of 26 subjects, and an expected mortality of 22%, as found by Sietsema et al.^5^. As a continuous variable, VO_2peak_ was not associated with mortality risk (HR 1.01; 95% CI 0.84−1.21).

## Discussion

We hypothesized that severely low CRF is associated with all-cause mortality risk in patients receiving HD. Although almost 35% of patients with severely low CRF died during the follow-up period, our results did not confirm our hypothesis, as there was no significant association between severely low CRF and all-cause mortality risk, even after adjustment for age, BMI, and HD vintage.

CRF has been described as a predictor of mortality in different populations, mainly to predict mortality from cardiovascular events^20,21,22^. Studies that evaluate the association between mortality and CRF using VO_2peak_ values in CKD patients are scarce in the literature. As far as we know, the studies performed by Sietsema et al.^5^ and Kohl et al.^23^ evaluated mortality risk associated with CRF assessed through CPET in patients receiving HD. Sietsema et al.^5^ evaluated 175 patients over 3.5 years. They found a significant association between CRF assessed through VO_2peak_ and mortality risk. A VO_2peak_ >17.5 mL∙kg^−1^∙min^−1^ was a powerful predictor of survival. In addition, age was an additional factor that significantly enhanced the predictive value^5^.

Sietsema et al.^5^ applied the median VO_2peak_ values as a cut-off to perform mortality analysis. We believe that the CRF of our subjects was worse than theirs because our VO_2peak_ median value was 14.1 mL∙kg^−1^∙min^−1^ and theirs was 17.5 mL∙kg^−1^∙min^−1^. Based on that and because of the ATS statement, we decided to diagnose our patients according to CRF severity^16^. According to the ATS statement, patients with a VO_2peak_ <15 mL∙kg^−1^∙min^−1^ have a physical disadvantage in performing activities that require physical effort. On the other hand, if VO_2peak_ is ≥15 mL∙kg^−1^∙min^−1^, the patient can perform physical effort comfortably. For this reason, Neder et al.^24^ considered that VO_2peak_ <15 mL∙kg^−1^∙min^−1^ is considered a severely low CRF and VO_2peak_ ≥15 mL∙kg^−1^∙min^−1^ is considered a mild-moderate CRF. Our results showed that those with severely low CRF had worse respiratory and cardiac performance, evaluated through ventilation and O_2_ pulse, respectively, as well as lower exercise tolerance on CPET, determined through CPET duration and final work rate. In addition, it is important to highlight that, although it was not statistically significant, patients with severely low CRF reported a lower frequency of exercise routine, an outcome that may influence the mortality rate^9^.

Interestingly, different from Sietsema et al.^5^, our findings did not indicate a significant association between severely low CRF and all-cause mortality. Similar to ours, Kohl et al.^23^ did not find a significant association between continuous VO_2peak_ values and mortality risk^23^. We believe that the lack of association may be due to the small sample size, which was also discussed by Kohl et al. as the main hypothesis. Cohort observational studies evaluating all-cause mortality may need larger sample sizes to achieve enough power in the statistical analysis, which may reduce the heterogeneity in the findings. Therefore, there is still a need for studies with a larger cohort size about the mortality risk and CRF.

In our analysis, there was a significantly higher prevalence of elderly and smoking habits in the severely low CRF group. Previous studies show that CRF may be influenced by age^7,25,26^. According to Imboden e al.^7^, CRF declines at about 3.5 mL∙kg^−1^∙min^−1^ (1-MET) per decade of age in healthy individuals. In addition, the pieces of evidence in smoking individuals were not enough to establish a relationship or causation between smoking and CRF^27^. However, it is known that the carbon monoxide from tobacco binds to red blood cells and reduces oxygen diffusion, which in the long-term may negatively impact CRF^27^.

Our study did not find significant results in continuous VO_2peak_ values and all-cause mortality risk. However, previous studies have demonstrated that higher CRF levels was associated with lower mortality risk, mainly in clinical populations^5,6,20,26^. Therefore, improvements in CRF in patients on HD may protective factor not only for mortality risk but also for comorbidities, such as cardiovascular diseases^20^. Our research group has been studying the effects of exercise on CRF and showed that combined exercise (i.e., aerobic and resistance) is beneficial in improving CRF in patients receiving HD^28,29^. In addition, as also recognized by Pella et al.^30^, the importance of a periodic evaluation of the maximum physical effort in this population must be recognized as a wide spectrum of health, as it is already routinely performed in other clinical populations (e.g., cardiac and chronic pulmonary patients).

Yet, our study has some limitations concerning sample selection, as participants were screened for a randomized clinical trial (n = 39) and only stable patients were included. The low power to detect mortality occurrence ratio in patients with severely low CRF may have been caused by our small sample size. Although all patients used beta-blocker medications and were instructed to maintain their routine medications (beta-blockers and vasodilators), we did not collect dosage or the active pharmaceutical ingredient or the use of vasodilators. Finally, we also did not collect the patients’ blood biochemistry and comorbidities beyond those related to CKD cause.

## Conclusion

We may conclude that severely low CRF and VO_2peak_ values were not associated with all-cause mortality in patients receiving HD. Although a severely low CRF prevailed in our sample, larger cohort studies are needed to establish strong conclusions on its association with all-cause mortality.
